# Preoperative detection of pleural adhesions by respiratory dynamic computed tomography

**DOI:** 10.1186/s12957-017-1280-7

**Published:** 2017-12-01

**Authors:** Junko Tokuno, Tsuyoshi Shoji, Ryota Sumitomo, Yuichiro Ueda, Keiji Yamanashi, Cheng-long Huang

**Affiliations:** Department of Thoracic Surgery; Kitano Hospital, The Tazuke Kofukai Medical Institute, Osaka, 530-8480 Japan

**Keywords:** Pleural adhesion, Dynamic CT, Preoperative evaluation, VATS

## Abstract

**Background:**

Video-assisted thoracic surgery (VATS) plays an important role in thoracic surgery because it is less invasive. However, the existence of severe pleural adhesions may make VATS difficult and complicated. The aim of this study was to assess the utility of inspiration and expiration computed tomography (respiratory dynamic CT (RD-CT)) in evaluation of pleural adhesions preoperatively.

**Methods:**

RD-CT was performed on 107 patients undergoing thoracotomies (both VATS and open). We assessed synchronous motion during respiration on RD-CT. Comparing the results of RD-CT and intraoperative findings, we assessed the utility of preoperative evaluation.

**Results:**

A negative correlation between sliding score and adhesion grade was revealed. Sliding score in adhesion negative patients was significantly higher than that in adhesion positive patients (*P* < 0.0001). The sensitivity of RD-CT was 63.6%, specificity was 74.1%, and accuracy was 72%. Among 62 patients with a CT-Respiration Ratio of less than 0.65, the sensitivity of RD-CT was 77.8%, specificity was 86.8%, and accuracy was 85.5%.

**Conclusions:**

RD-CT may be clinically useful for detecting the presence of pleural adhesions. It can be adopted as one of the criteria for deciding the surgical approach.

## Background

Due to the development of surgical techniques and tools, video-assisted thoracoscopic surgery (VATS) has become an important approach for thoracic surgery for the lungs, as it is less invasive, and it has gained widespread acceptance. But pleural adhesions between visceral and parietal pleura could increase the risk of lung injury and may require conversion to open surgery in cases with severe pleural adhesions, and it may result in prolongation of the operation time on VATS [1, 2]. Therefore, preoperative detection of pleural adhesions would be useful for assessing the surgical approach.

The presence of pleural adhesions is expected in cases of inflammatory disease in the pleural cavity, such as pneumonia, lung abscess, pyothorax, and tuberculosis. Some studies have evaluated pleural adhesions preoperatively by conventional chest computed tomography (CT) [3] or ultrasonography [4–7]. There are also some reports describing the usefulness of respiratory dynamic (RD) CT [8, 9] or magnetic resonance imaging (MRI) [10–12] for detecting chest wall or aortic invasion by lung cancer. They evaluated the tumor location and its motion during respiration comparing the lung and chest wall or aorta. We hypothesized that lungs move independently from the chest wall when there are no pleural adhesions, and the movements of lungs are limited when there are pleural adhesions. The aim of this study was to assess the utility of RD-CT for detecting pleural adhesions preoperatively.

## Methods

### Patients

This study was designed as a prospective study, and the institutional review board of our institution gave its approval (P14-06-017). From January to December 2014, excluding cases treated with urgent surgery, cases without thoracotomy and cases with pneumothorax, in total, 107 patients met the criteria of this study and informed consent was obtained from each patient. The characteristics of the 107 patients are shown in the Supplemental table. There were 59 men and 48 women, with a mean age of 65.8 years (range from 30 to 84). Seventy-eight patients had primary lung cancer, 14 patients had metastatic lung tumor, and 3 patients had mediastinal tumor. Among these 107 patients, intraoperative findings regarding the presence and/or absence of pleural adhesions could be completely confirmed. In addition, respiratory functions were also recorded in all patients.

### CT technique and imaging analysis

Setting the patients in supine position, CT (Aquillion, Toshiba Medical Systems, Tochigi) scans were performed during each of the inspiration and expiration phases. The X-ray tube voltage was 120 kV, and the tube current was 200 mA for the inspiration phase and 100 mA for the expiration phase. The section thickness was 0.5 or 1 mm for the inspiration phase and 1 mm for the expiration phase.

**Fig. 1:**
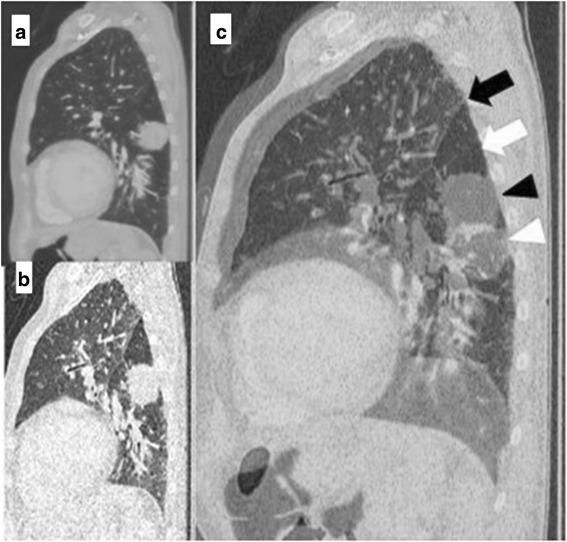
Images of a case of no pleural adhesion (adhesion grade 0). **a** Sagittal CT image of inspiration phase. **b** Sagittal CT image of expiration phase. **c** Two images superimposed. Significant fissure lines and tumor movements were seen. White arrow indicates fissure line in inspiration phase. White arrow head indicates a tumor in inspiration phase. Black arrow indicates fissure line in expiration phase. Black arrow head indicates a tumor in expiration phase

**Fig. 2:**
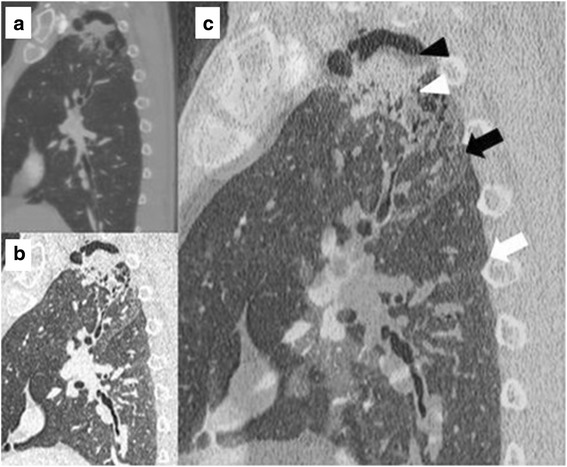
Images of a case of partial strong pleural adhesion (adhesion grade 2). **a** Sagittal CT image of inspiration phase. **b** Sagittal CT image of expiration phase. **c** Two images superimposed. Tumor was fixed during two phases of inspiration. White arrow indicates fissure line in inspiration phase. White arrow head indicates a tumor in inspiration phase. Black arrow indicates fissure line in expiration phase. Black arrow head indicates a tumor in expiration phase

All the images were analyzed by three-dimensional CT image software, SYNAPSE VINCENT (Fujifilm Medical, Tokyo). The two images (inspiration phase and expiration phase) were superimposed on SYNAPSE VINCENT. We preferred the sagittal images because these images could be assessed more conveniently than axial and coronal ones (Figs. 1 and 2). Superimposing the images, we assessed the motion of the lung by three points: the front edge of the fissure line, the dorsal edge of the fissure line, and the pulmonary lesion in each case. With respect to RD-CT findings, we classified the sliding grade as four scores: 0 (totally fixed), 1 (movement within the same rib or the same intercostal space), 2 (movement under one intercostal space), and 3 (movement over one intercostal space). And we gave the sliding score to each case in the sagittal image slide which the biggest motion of fissure line on the chest wall was seen.

The sums of grades in three points were calculated as sliding scores. This evaluation was performed by thoracic surgeons.

Furthermore, in order to evaluate whether the patients could expire sufficiently in expiratory phase in RD-CT, we also estimated the lung volume at RD-CT, both the inspiration phase and expiration phase, and calculated the ratio of the expiration volume by the inspiration volume (CT-Respiratory Ratio), using the SYNAPSE VINCENT. As a result, 62 patients had less than 0.65 of CT-Respiratory Ratio.

**Fig. 3:**
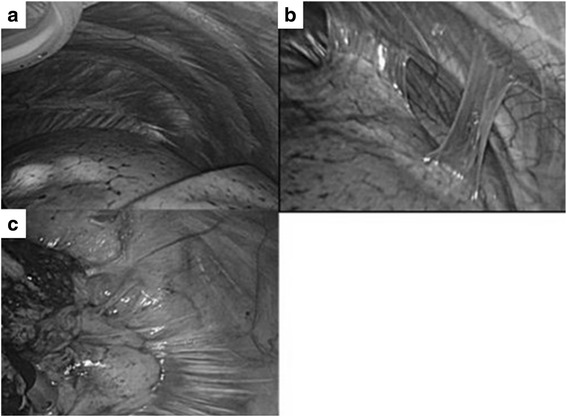
Representative image of adhesion grade. **a** No adhesion (adhesion grade 0). **b** Only band adhesion (adhesion grade 1). **c** Partial but strong adhesion (adhesion grade 2)

Regarding the severity of pleural adhesion, the adhesion grade was categorized into four grades as follows: 0 (no adhesion) (Fig. 3a), 1 (only band adhesion without surgical disturbance) (Fig. 3b), 2 (soft membranous adhesion in partial portion) (Fig. 3c), and 3 (tight and strong adhesion all over the lung). Cases with 0 to 1 of adhesion grade were classified adhesion negative, and cases with 2 to 3 of adhesion grade were classified as adhesion positive.

### Statistical analysis

A Pearson’s correlation coefficient was used to evaluate relationships between sliding grade and adhesion grade. In addition, regarding the sliding score, we classified 107 patients into two groups: patients with less than 6 of sliding score (RD-CT positive), and patients with sliding score 6 and more (RD-CT negative) because this cutoff value showed the strongest statistical significance in relation to adhesion grade.

Furthermore, with respect to intraoperative findings, 107 patients were divided into two groups: patients with adhesion grade 0–1, and patients with adhesion grade 2–3, owing to the selection for surgical procedures.

Comparisons of the preoperative RD-CT findings and the intraoperative adhesion grade were estimated using the *t* test and the *χ* ^2^. A *P* value of < 0.05 was considered to be significant in all statistical analysis. Then, we calculated the sensitivity, specificity, and accuracy of RD-CT.

## Results

Among the 107 patients studied, 77 cases (72%) were accomplished by VATS, and conversion to open surgery was required in 4 cases (3.7%). The reason for the conversion was intraoperative hemorrhage of all four cases. Clinical node positive cases and cases with bronchoplasty underwent open surgery.

Concerning the intraoperative findings, 69 patients were adhesion grade 0, 18 patients were adhesion grade 1, 13 patients were adhesion grade 2, and 7 patients were adhesion grade 3.

Average operation time for lobectomy were as follows: In cases without severe adhesions (adhesion grade 0 or 1), 239.6 ± 44.5 min by open surgery, by 204.2 ± 35.8 min VATS. In cases with severe adhesions (adhesion grade 2 or 3), 284.6 ± 91 min by open surgery, 215.6 ± 47.9 min by VATS. No significant difference between both groups with or without severe adhesions was observed (*p* = 0.04, 0.22 by open surgery, VATS, respectively).

Regarding the front edge of the fissure, 3 patients were sliding grade 0, 23 patients were sliding grade 1, 52 patients were sliding grade 2, and 29 patients were sliding grade 3. No significant relationship was found between the sliding grade of the front edge of the fissure and the adhesion grade by *t* test.

With respect to the dorsal edge of the fissure, 5 patients were sliding grade 0, 20 patients were sliding grade 1, 42 patients were sliding grade 2, and 40 patients were sliding grade 3. No significant relationship was also observed between the sliding grade of the dorsal edge of the fissure and the adhesion grade by *t* test.

Regarding the specific lesion in each case, 8 patients were sliding grade 0, 22 patients were sliding grade 1, 28 patients were sliding grade 2, and 49 patients were sliding grade 3. No significant relationship was found between the sliding grade of the specific lesion and the adhesion grade by *t* test.

The sliding scores and the adhesion grades in all 107 patients are shown in Table 1. Regarding the sliding score, among all 107 patients studied, 36 patients were less than 6 of sliding score, 18 patients were sliding score 6, 23 patients were sliding score 7, 18 patients were sliding score 8, and 12 patients were sliding score 9. A significant negative correlation between sliding score and adhesion grade was revealed (*r* = − 0.473, *P* < 0.0001). The sliding scores were 6.58 ± 1.74 in adhesion negative patients and 4.68 ± 2.06 in adhesion positive patients. Sliding score was significantly higher in adhesion negative patients than in adhesion positive patients (*P* < 0.0001). Among the 107 patients regarding the relationships between RD-CT findings and intraoperative findings, 14 cases were true positive, 63 cases were true negative, 22 cases were false positive, and 8 cases were false negative (Table 2). In total, sensitivity was 63.6%, specificity was 74.1%, and accuracy was 72%.

**Table 1:** Distributions of sliding score and adhesion grade in all 107 patients

SS	< 6	6	7	8	9
AG 0	14	15	17	13	10
AG 1	8	2	3	3	2
AG 2	7	1	3	2	0
AG 3	7	0	0	0	0

*SS* sliding score, *AG* adhesion grade

**Table 2:** Distributions of RD-CT evaluation and intraoperative findings in all 107 patients

	Adhesion seen	
	Yes	No
Adhesion predicted		
Yes	14	22
No	8	63

Furthermore, we performed an additional evaluation using 62 patients with a CT-Respiratory Ratio of less than 0.65. Regarding the sliding score, 14 patients were sliding score less than 5, 10 patients were 6, 15 patients were 7, 12 patients were 8, and 11 patients were 9. adhesion grade distribution was as follows: 43 patients were adhesion grade 0, 10 patients were 1, 4 patients were 2, and 5 patients were 3 (Table 3). As a result, among patients with a CT-Respiratory Ratio of less than 0.65, the sliding score significantly negatively correlated with adhesion grade (*r* = − 0.633, *P* < 0.0001). The sliding scores were 7.17 ± 1.95 in adhesion negative patients and 3.89 ± 1.76 in adhesion positive patients. Sliding score was significantly higher in adhesion negative patients than in adhesion positive patients (*P* < 0.0001). Regarding the RD-CT findings and intraoperative findings among patients with a CT-Respiratory Ratio of less than 0.65, the present study showed 8 true positive, 37 true negative, 16 false positive, and 1 false negative (Table 4). It revealed sensitivity was 77.8%, specificity was 86.8%, and accuracy was 85.5%.

**Table 3:** Distributions of sliding score and adhesion grade in all 62 patients

SS	< 6	6	7	8	9
AG 0	4	8	11	11	9
AG 1	2	1	3	1	2
AG 2	3	1	1	0	0
AG 3	5	0	0	0	0

*SS* sliding score, *AG* adhesion grade

**Table 4:** Distributions of RD-CT evaluation and intraoperative findings in 62 patients with a CT-Respiration Ratio of less than 0.65

	Adhesion seen	
	Yes	No
Adhesion predicted		
Yes	7	7
No	2	46

Finally, we performed an evaluation of the correlation between two points (front and dorsal fissure edges) and pleural adhesions. However, no significant relationship was found between this evaluation and adhesion grade (data not shown).

## Discussion

In cases with severe pleural adhesion, thoracic surgery for the lungs would be complicated and risks would be enlarged. This is particularly the case with VATS lobectomy, which is widely performed for lung cancer patients. For example, Marty-Ane et al. reported that conversion to open surgery occurred in 6.1% among 410 cases of VATS lobectomy and that pleural adhesion was one of the main reasons along with hemorrhage and extension of the tumor [1]. In addition, Puri et al. analyzed 87 cases of VATS lobectomy that required conversion to open thoracotomy, and they reported that 59.8% of cases were converted due to pleural adhesions [2]. Therefore, if we could detect the pleural adhesion preoperatively, we could not only construct plans for surgical approach but also prepare more precisely for the risks, such as transfusions and giving proper information to patients preoperatively.

There have been several studies on preoperative evaluations for pleural adhesions. Mason et al. reported that conventional CT scans had limited accuracy to detect pleural adhesions [3]. On the other hand, there have been several studies about the prediction of pleural adhesions by ultrasonography [4–7]. Although ultrasonography is a noninvasive examination, this examination requires well-trained testers and is much more time consuming. Therefore, we have been trying to find a simple and more convenient examination to detect pleural adhesions. In particular, evaluation by CT image seems to have more objectivity and reproducibility than that by ultrasonography. In addition, CT has been routinely required for preoperative evaluation even when we do not intend to detect pleural adhesions.

In fact, several studies have previously reported the usefulness of respiratory dynamic (RD)-CT [8, 9] and RD-MRI [10–13] for detecting the tumor invasion to the chest wall or aorta preoperatively. For example, Shiotani et al. [13] stated that the RD-MRI was useful for evaluation of chest wall invasion of the tumor. Therefore, we considered that the RD-CT might be useful to detect the pleural adhesion preoperatively for lung surgery. In general, patients undergoing operation would be preoperatively evaluated by CT, in the inspiration phase. Only images of the expiration phase were simultaneously added in the RD-CT. The radical dosage in the expiration phase could be decreased lower than that of a usual inspiration image without any difficulty for evaluation of the movements of the lungs. Furthermore, the construction of 3D CT imaging was performed by SYNAPSE VINCENT, in order to evaluate the RD-CT more precisely.

In the RD-CT, we evaluated three points, the front edge of the fissure, the dorsal edge of the fissure, and the specific lesion in each case. Consequently, the present study revealed that the sliding score using three points was available to evaluate severe pleural adhesions preoperatively. The sliding score using three points was significantly higher in adhesion negative patients than in adhesion positive patients. In contrast, no significant relationship was found in either evaluation using one point or evaluation using two points. These results might be partly due to the limited number of cases in the present study.

For detecting cases with adhesion grade 2 or 3 in all 107 patients, the finding of the RD-CT showed a sensitivity of 63.6%, specificity of 74.1%, and accuracy of only 72%. However, for detecting cases with adhesion grade 3, the finding of the RD-CT demonstrated a sensitivity of 100% in all 107 patients. Therefore, reduction of the number of false positives is critical for the clinical application of RD-CT.

Considering these circumstances, the CT-Respiration Ratio was proven to be important for accurate evaluation in the RD-CT. By analyzing the 3D CT using SYNAPSE VINCENT, we could easily calculate the lung volume, both in the inspiration phase and in the expiration phase. To find the most appropriate cutoff, we examined by setting CT-Respiratory Ratio 0.60, 0.65, and 0.70, respectively. Setting CT-Respiratory Ratio 0.65 as cutoff (*n* = 62), the sensitivity was 77.8% and the specificity was 86.8%. Setting CT-Respiratory Ratio 0.70 as cutoff (*n* = 78), the sensitivity was 72.3% and the specificity was 82.1%. And setting CT-Respiratory Ratio 0.60 as cutoff (*n* = 46), the sensitivity was 100% and the specificity was 90%. The accuracy was highest when we set CT-Respiratory Ratio 0.60 as cutoff, but the number of the patients was so limited that we finally found CT-Respiratory Ratio 0.65 the most suitable one.

As a result, a stronger association between the sliding scale and the adhesion grade was observed among patients with small CT-Respiration Ratio who could expire efficiently. In contrast, most of the false positive cases were due to inefficient respiration, indicated by the large CT-Respiration Ratio. Therefore, proper practice for holding in the expiration phase is considered to be important to reduce the number of false positive cases. In fact, the present study revealed that there was no correlation between CT-Respiratory Ratio and FEV1.0% (*r* = − 0.141), or between CT-Respiratory Ratio and %VC (*r* = − 0.148) or adhesion grade and %VC (*r* = − 0.054). RD-CT seems to be independent of respiration function.

Concerning the conversion from VATS to open surgery, as we mentioned above, the presence of adhesions seems to be one of the main reasons for conversion; there was no case of conversion due to adhesion in this study. Eight cases (42.1% of the cases with severe adhesions) underwent open surgery from beginning because of technical reason such as bronchoplasty or node positive and so on, so the number of conversion was limited as a result, while cases underwent open surgery from beginning in the 18 cases (20.7% of the cases without severe adhesions).

Moreover, regarding operation time, no significant differences between with and without severe adhesions were revealed maybe because certain cases with severe adhesions were performed by open surgery so operation time may have been relatively shortened. Although there were no significant differences, operation time tended to be longer in the cases with severe adhesion in both open and VATS. Considering these results, it seems to help plan the operation schedule and give the patients prompt information preoperatively.

There are some limitations to this study. Firstly, the ratio of false positive cases was higher than those we expected. This may be due to inefficient respiration, as mentioned above, and a chest wall tumor. Concerning insufficient respiration, it seems to be important to practice holding expiration before RD-CT. Secondly, evaluation points in the RD-CT may be limited. We set the edges of the fissure and specific lesions as “milestones” in this study. Therefore, the evaluation for pleural adhesion which locally existed apart from these points, for example, mediastinum and hilum in which VATS procedures are more challenging than other areas, might be less accurate however severe the adhesion is, and it might relate to false negative. Actually, in this study we excluded the cases of mediastinal tumor. Regarding the apex, although the lung movements and sliding also had seemed to be limited than parietal area, we successfully predicted adhesion at the apex showed in Fig. 2. In our perspective, parietal pleural adhesions were usually due to some previous inflammation in thoracic cavity. Inflammation sometimes spreads to the whole hemithorax; therefore, mediastinum or hilum adhesions were often observed when we found parietal pleural adhesions. Existing of parietal pleural adhesion may connect to mediastinum and hilum adhesions. With this idea, we put weight on to detect parietal pleural adhesions. Thirdly, this is a single institution study with a relatively small number of patients. Fourthly, to exam expiration phase, CT scan needed additional irradiation. To minimalize the irradiation, we reduced the tube current in expiration phase. With respect to expecting the operation time, avoiding intraoperative lung injury, RD-CT is still beneficial.

Although the issue still remains about false positives, the method of RD-CT has a sensitivity of 100% and identify the absence of severe pleural adhesions.

## Conclusions

We think that RD-CT is a clinically useful preoperative examination for evaluating pleural adhesions for lung surgery cases.
